# The Modulation of Regulatory T Cells via HMGB1/PTEN/β-Catenin Axis in LPS Induced Acute Lung Injury

**DOI:** 10.3389/fimmu.2019.01612

**Published:** 2019-07-25

**Authors:** Min Zhou, Haoshu Fang, Min Du, Changyong Li, Rui Tang, Haiyan Liu, Zhi Gao, Zongshu Ji, Bibo Ke, Xu-Lin Chen

**Affiliations:** ^1^Neurocritical Care Unit, The First Affiliated Hospital of USTC, Division of Life Sciences and Medicine, University of Science and Technology of China, Hefei, China; ^2^Department of Neurosurgery, The First Affiliated Hospital of USTC, Division of Life Sciences and Medicine, University of Science and Technology of China, Hefei, China; ^3^Department of Pathophysiology, Anhui Medical University, Hefei, China; ^4^Department of Physiology, Wuhan University School of Basic Medical Sciences, Wuhan, China; ^5^Department of Surgery, The Dumont-UCLA Transplant Center, David Geffen School of Medicine at UCLA, Los Angeles, CA, United States; ^6^Department of Burns, The First Affiliated Hospital of Anhui Medical University, Hefei, China

**Keywords:** acute lung injury, HMGB1, regulatory T cells, sepsis, inflammation

## Abstract

Sepsis-induced acute lung injury (ALI)/acute respiratory distress syndrome (ARDS) remains the leading complication for mortality caused by bacterial infection. The regulatory T (Treg) cells appear to be an important modulator in resolving lung injury. Despite the extensive studies, little is known about the role of macrophage HMGB1/PTEN/β-catenin signaling in Treg development during ALI.

**Objectives:** This study was designed to determine the roles and molecular mechanisms of HMGB1/PTEN/β-catenin signaling in mediating CD4^+^CD25^+^Foxp3^+^ Treg development in sepsis-induced lung injury in mice.

**Setting:** University laboratory research of First Affiliated Hospital of Anhui Medical University.

**Subjects:** PTEN/β-catenin Loxp and myeloid-specific knockout mice.

**Interventions:** Groups of PTEN^loxp^/β-catenin^loxp^ and myeloid-specific PTEN/β-catenin knockout (PTEN^M−KO^/β-catenin^M−KO^) mice were treated with LPS or recombinant HMGB1 (rHMGB1) to induce ALI. The effects of HMGB1-PTEN axis were further analyzed by *in vitro* co-cultures.

**Measures and Main Results:** In a mouse model of ALI, blocking HMGB1 or myeloid-specific PTEN knockout (PTEN^M−KO^) increased animal survival/body weight, reduced lung damage, increased TGF-β production, inhibited the expression of RORγt and IL-17, while promoting β-catenin signaling and increasing CD4^+^CD25^+^Foxp3^+^ Tregs in LPS- or rHMGB-induced lung injury. Notably, myeloid-specific β-catenin ablation (β-catenin^M−KO^) resulted in reduced animal survival and increased lung injury, accompanied by reduced CD4^+^CD25^+^Foxp3^+^ Tregs in rHMGB-induced ALI. Furthermore, disruption of macrophage HMGB1/PTEN or activation of β-catenin significantly increased CD4^+^CD25^+^Foxp3^+^ Tregs *in vitro*.

**Conclusions:** HMGB1/PTEN/β-catenin signaling is a novel pathway that regulates Treg development and provides a potential therapeutic target in sepsis-induced lung injury.

## Introduction

Sepsis is a systemic inflammatory response syndrome which may result in acute lung injury (ALI) and/or acute respiratory distress syndrome (ARDS) (1). ARDS is a type of respiratory failure characterized by rapid onset of widespread inflammation in the lungs, symptoms include shortness of breath, rapid breathing, and bluish skin coloration (2). Despite recent progress in developing many pharmacological interventions for ALI/ARDS, there have been no successful clinical trials for drugs treating these disorders, implying that there are complex molecular mechanisms in sepsis-driven inflammatory responses.

High-mobility group box 1 protein (HMGB1), a highly conserved and ubiquitous DNA binding nuclear protein, is a key mediator during inflammatory responses in sepsis (3). HMGB1, as an innate “danger signal” (alarmin), plays a key role in the initiating innate and adaptive immune response (4–6). As a late mediator, HMGB1 can be actively released from endotoxin-stimulated macrophages following lipopolysaccharide (LPS) and by TNF-α or IL-1β stimulation. Blockade of HMGB1 via antibody targeting protects against LPS lethality in mice, whereas administration of HMGB1 in mice results in developing endotoxemia and lethality (7). HMGB1 contributes to the endotoxin-induced ALI through activating NF-κB translocation, increasing levels of proinflammatory cytokines, and enhancing lung permeability (8–10). Extracellular HMGB1 augmented autoimmune response through stimulating dendritic cell maturation and macrophage activation, whereas HMGB1 deficiency resulted in increasing the number of lymph node CD4^+^Foxp3^+^ regulatory T (Treg) cells during inflammatory response (11). Moreover, disruption of HMGB1 promotes the ability to induce Treg and enhances antitumor immunity (12).

Recently, CD4^+^CD25^+^Foxp3^+^ Tregs have been shown to be crucial for the resolution of endotoxin-induced lung injury via both TGF-β-dependent and -independent pathways (13). TGF-β induces Treg-mediated suppressive activity and Foxp3 expression (14, 15). The development and survival of CD4^+^CD25^+^ Tregs *in vivo* was depressed by the increased phosphatase and tensin homolog deleted on chromosome ten (PTEN) activity via distinct IL-2 receptor (IL-2R) signaling, which is associated with downstream mediators of PI3K (16). Deficiency of myeloid PTEN increases PI3K signaling and reduces endotoxin-induced inflammatory response and lung injury (17). Indeed, loss of PTEN leads to an increasing nuclear accumulation of β-catenin (18) and promotes PI3K, which P3 and activates downstream PDK1 and Akt (19). Increasing phosphorylation of Akt by PDK1 enhances Akt activity and facilitates Treg induction (20), whereas deletion of PDK1 in T cells results in reducing Treg numbers *in vitro* and *in vivo* (21). Thus, the modulation of Treg development might involve in multiple pathways during lung inflammation and injury.

Using a well-established model of lung injury and an *in vitro* co-culture system, we identified a novel regulatory pathway of HMGB1/PTEN/β-catenin signaling on Treg induction during inflammatory response. We demonstrated that HMGB1 promoted lung inflammation through activating myeloid PTEN-mediated innate immunity. Lacking myeloid PTEN ultimately resulted in promoting β-catenin activation and TGF-β production, which in turn induced CD4^+^CD25^+^Foxp3^+^ Tregs and suppressed endotoxin-mediated inflammation in the lung. Our data document that HMGB1/PTEN/β-catenin signaling is critical for development of Tregs in the resolution of sepsis-induced lung injury.

## Materials and Methods

### Mice

The floxed β-catenin (β-catenin^flox^) mice (The Jackson Laboratory, Bar Harbor, ME), and the mice expressing Cre recombinase under the control of the Lysozyme M (LysM) promoter (LysM-Cre; The Jackson Laboratory) were used to generate myeloid-specific β-catenin knockout (β-catenin^M−KO^) mice. In brief, homozygous β-catenin^flox^ mice were interbred with homozygous LysM-Cre mice, and the heterozygous offspring were then backcrossed to the homozygous β-catenin^flox^ mice to generate β-catenin^M−KO^ (LysM-Cre-β-catenin^flox^) mice. The C57BL/6 wild-type (WT) and PTEN^flox^ mice were purchased from The Jackson Laboratory (Bar Harbor, ME). The expression of β-catenin was detected in spleen and myloid cells, respectively (Figure S1). The myeloid-specific PTEN knockout (PTEN^M−KO^) mice were generated as described (22). Mouse genotyping was performed by using a standard protocol with primers described in the JAX Genotyping protocols database, and the expression of PTEN was detected as described (22). All animals were housed in animal facility under specific pathogen-free conditions. Animals at 8–10 weeks of age were used in all experiments.

### Mice Treatment

To establish the animal model of ALI, mice were anesthetized with i.p. ketamine (150 mg/kg) and acetylpromazine (13.5 mg/kg), and then an incision (1–2 cm) was made on the animal neck to expose the trachea. A 20-gauge catheter was inserted into the lumen of trachea. 50 μl of LPS (*Escherichia coli* 055:B5; Sigma-Aldrich, 100 μg/mouse), diluted in sterile water was instilled via the catheter. Sterile water was used in the control group (8–10 mice per group) (13). To determine the role of HMGB1 during LPS-induced ALI, mice were instilled with 100 μg/mouse of anti-HMGB1 (Product# 326052233, Shino-TEST Co, Tokyo, Japan) immediately after LPS instillation. Control mice received the same volume of saline solution or control IgG (Sigma-Aldrich). To generate mouse model of endotoxin-induced sepsis, mice were injected with LPS (750 μg/mouse, i.p.) as described (23). In some experiments, mice were administrated with recombinant HMGB1 (rHMGB1, 50 μg/mouse, i.p., product# 4652, Sigma-Aldrich) or vehicle PBS. Since previous reports showed that maximal lung injury and HMGB1 expression occurred between 12 and 48 h after LPS instillation (24), all animal studies were executed at 24 h after LPS, rHMGB1, anti-HMGB1, control IgG or saline treatment.

### Analysis of the Permeability Index

The permeability index, reflexing the damage of alveolar epithelial and endothelial permeability, was evaluated by administrating human serum albumin (i.v. 25 μg; Signa-Aldrich, MO) 1 h prior to sacrificing the animal. The blood and BALF were collected at the time of sacrifice. ELISA assay was performed to measure the level of human albumin concentration using a human serum albumin ELISA kit (Cayman Chemical, Ann Arbor, MI). The pulmonary permeability index was defined as the human albumin concentration in BAL fluid/serum ratio.

### Analysis of Bronchoalveolar Lavage Fluid (BALF)

The mice were anesthetized before exposure of the trachea. After the catheter was inserted into the lumen of trachea, the lungs were then lavaged 3 times with 0.8 ml of sterile saline. The total collected lavage averaged 1.4–1.7 ml/mouse. BALF was centrifuged at 800 × g for 10 min at 4°C. The cell-free supernatants were stored at −80°C for later analysis. The cell pellet was re-suspended in PBS and counted by a hemacytometer. The differential staining was performed with Diff-Quik staining solutions to count enriched alveolar macrophages as described (25).

### Analysis of HMGB1 and Cytokines

The mouse ELISA kits were used to measure the levels of HMGB1 (Shino-TEST Co, Tokyo, Japan), TGF-β, TNF-α, IL-1β, IL-17A, and IL-23 (p19) (eBioscience) in BALF, serum and co-cultures according to the manufacturer's instructions.

### Histological Analysis

The lungs from mice (*n* = 8/group) were harvested and rinsed with PBS, and then immersed into 10% of buffered formalin overnight. After processing for paraffin embedding, the lung sections were stained with hematoxylin and eosin (H&E). The severity of lung injury was evaluated semi-quantitatively by grading score on a scale from 1 to 5 as described (13). In this classification, 1, normal; 2, focal (<50% lung section) interstitial congestion and inflammatory cell infiltration; 3, diffuse (>50% lung section) interstitial congestion and inflammatory cell infiltration; 4, focal (<50% lung section) consolidation and inflammatory cell infiltration; 5, focal (>50% lung section) consolidation and inflammatory cell infiltration. The mean score was determined by examining each sample.

### Myeloperoxidase Activity Assay

The presence of myeloperoxidase (MPO) was used as an index of lung neutrophil accumulation as described (26). The frozen tissue samples were homogenized and separated by centrifugation. Supernatants were analyzed for MPO activity by spectrophotometry at 655 nm, and the change in absorbance was measured. One unit of MPO activity was defined as the quantity of enzyme degrading 1 μmol peroxide/min at 25°C per gram of tissue.

### Western Blot Analysis

Protein was extracted from macrophages with ice-cold protein lysis buffer (50 mM Tris, 150 mM Nacl, 0.1% sodium dodecyl sulfate, 1% sodium deoxycholate, 1% Triton-100). The buffer contains 1% proteinase and phosphatase inhibitor cocktails (Sigma-Aldrich). Proteins (30 μg/sample) in SDS-loading buffer (50 mM Tris, pH 7.6, 10% glycerol, 1% SDS) were subjected to SDS-polyacrylamide gel electrophoresis (PAGE) and transferred to nitrocellulose membrane (Bio-Rad, Hercules, CA). The membrane was blocked with 5% dry milk and 0.1% Tween 20 (USB, Cleveland, OH). Monoclonal rabbit anti-mouse HMGB1 (product# 6893), PTEN (product# 9188), β-catenin (product# 8480), phos-PDK1 (product# 3438), phos-Akt (ser473) (product# 4060), and β-actin (product# 3700) Abs (Cell Signaling Technology, MA) were used. The membranes were incubated with Abs, and then developed according to the Pierce SuperSignal West Pico Chemiluminescent Substrate protocol (Pierce Biotechnology, Rockford, IL). Relative quantities of protein were determined and expressed in absorbance units (AU) comparing to β-actin expression using a densitometer (Kodak Digital Science 1D Analysis Soft-ware, Rochester, NY).

### Quantitative RT-PCR Analysis

Total RNA was purified from lung tissue, peripheral blood or spleen T cells using RNeasy Mini Kit (Qiagen, Chatsworth, CA) according to the manufacturer's instructions. Reverse transcription to cDNA was performed by using SuperScript III First Strand Synthesis System (Invitrogen). Quantitative real-time PCR was performed using the DNA Engine with Chromo 4 Detector (MJ Research, Waltham, MA). In a final reaction volume of 25 μl, the following were added: 1 × SuperMix (Platinum SYBR Green qPCR Kit; Invitrogen, San Diego, CA) cDNA and 10 μM of each primer. Amplification conditions were: 50°C (2 min), 95°C (5 min), followed by 40 cycles of 95°C (15 s) and 60°C (30 s). Primer sequences used for the amplification of TNF-α, TGF-β, IL-17A, IL-23, RORγt, Foxp3, and HPRT are shown in Supplementary Table 1. Target gene expressions were calculated by their ratios to the housekeeping gene HPRT.

### Cell Isolation

The WT, PTEN^flox^, PTEN^M−KO^, β-catenin^flox^, and β-catenin^M−KO^ mice were anesthetized with sodium pentobarbital (100 mg/kg, i.p.), and then Bio-Gel elicited peritoneal macrophages were isolated as described previously (22). The macrophages were cultured in medium (Invitrogen) supplemented with 10% FBS, 100 μg/ml of penicillin/streptomycin (Life Technologies; Grand Island, NY). The peripheral blood or spleen T cells were purified using the EasySep™ mouse T cell isolation kit (STEMCELL Technologies, Vancouver, BC, Canada) according to the manufacturer's instructions. T cells were then stimulated with anti-CD3 (1 μg/ml, Clone 145-2C11) and anti-CD28 (2 μg/ml, Clone 37.51) (eBioscience).

### *In vitro* Transfection and Treatments

After 24 h cell culture, 1 ×10^6^ macrophages/well were transfected with 100 nM of HMGB1 siRNA or non-specific control siRNA using lipofectamine 2000 reagent (Invitrogen), and incubated for 24 h. Non-specific (NS) siRNA as a control. In some experiments, cells were pretreated with 10 μg/ml of rHMGB1 or 10 μg/ml of anti-HMGB1 for 24 h, and then were supplemented with 1 μg/ml of LPS for additional 6 h. The HMGB1 siRNA and control siRNA were purchased from Santa Cruz Biotechnologies (Santa Cruz, CA).

### Macrophage/T Cell Co-cultures

The HMGB1 siRNA-transfected macrophages or macrophages isolated from WT, PTEN^flox^, PTEN^M−KO^, β-catenin^flox^, and β-catenin^M−KO^ mice were suspended at 5 ×10^5^ cells/ml and cultured on 60 mm plates. After the cells were stimulated with LPS (1 μg/ml) for 6 h, spleen T cells were then added into cultures at a macrophage/T cell ratio of 1:10 as described before (27). The co-cultured cells were incubated for 24 h, and then macrophages and spleen T cells were harvested for the Western blots, real-time PCR, and flow cytometry analysis.

### Flow Cytometry Analysis

Peripheral blood T cells isolated from LPS- and/or anti-HMGB1-treated WT, or rHMGB1-treated PTEN^flox^, PTEN^M−KO^, β-catenin^flox^, and β-catenin^M−KO^ mice, as well as spleen T cells harvested from co-cultures were stained with anti-mouse CD4-PE-Cyanine5 (RM4-5), CD25-PE (PC61.5), and Foxp3-FITC (FJK-16s) mAbs (eBioscience) according to the manufacturer's instructions. PE-labeled rat anti-mouse IgG2a isotypes were used as negative controls. Measurements were performed using a FACSCalibur flow cytometer (BD Biosciences). Data analysis was performed using CellQuest software.

### Statistical Analysis

All experiments were repeated three times. Data are expressed as mean±SD and analyzed by Permutation *t*-test and Pearson correlation. Per comparison two-sided *p*-values <0.05 were considered statistically significant. Multiple group comparisons were performed using one-way ANOVA with the *post-hoc* test. The body weight loss was analyzed by using student's *t*-test. All analyses were made using SAS/STAT software, version 9.4.

## Results

### Blocking HMGB1 Ameliorates Lung Damage, Increases TGF-β Production, and Suppresses Proinflammatory Mediators in Acute Lung Injury

**Figure 1:**
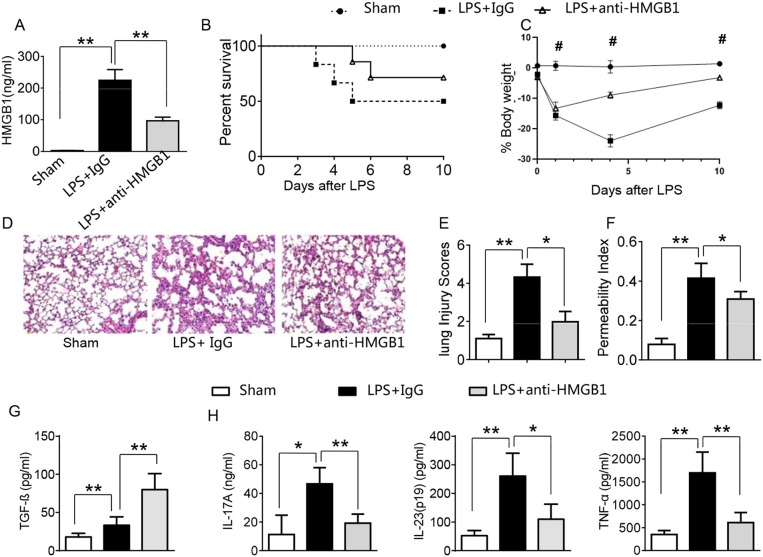
Blocking HMGB1 ameliorates lung damage, increases TGF-β production, and suppresses proinflammatory mediators in acute lung injury. Mice were subjected to LPS or anti-HMGB1 instillation via a catheter after exposure of the trachea. **(A)** HMGB1 levels were measured by ELISA assay in BAL fluid from sham, LPS-stilled, and anti-HMGB1 antibody-treated mice. Mean±SD (*n* = 4–6 samples/group), ^**^p < 0.01. **(B)** Animal survival (*n* = 8 animals per group). **(C)** Body weight (*n* = 8). **(D)** Lung sections were stained with H&E. Original magnification, ×40; **(E)** Histopathological mean lung injury scores (*n* = 8 animals per group); ^**^p < 0.01. **(F)** The pulmonary permeability index was measured in sham, LPS-instilled, and anti-HMGB1 antibody-treated mice (*n* = 8 animals per group); ^**^p < 0.01. ELISA assay was performed in BAL fluid from sham, LPS-stilled, and anti-HMGB1 antibody-treated mice **(G)** TGF-β, **(H)** IL-17A, IL-23 (p-19), and TNF-α; Mean ± SD (*n* = 4-6 samples/group), ^*^p < 0.05, ^**^p < 0.01, ^#^*p* < 0.05 between LPS + IgG and LPS+anti-HMGB1 group.

LPS has been shown to induce HMGB1 release and triggers systemic inflammatory response in sepsis (3, 13, 23). Using the mouse model of LPS-induced ALI, we found that instillation of LPS significantly increased HMGB1 levels in BALF compared to sham controls (Figure 1A, 224.6 ± 33.7 vs. 2.6 ± 0.33, *p* <0.01). In contrast, neutralized HMGB1 release with polyclonal anti-HMGB1 treatment reduced HMGB1 levels (96.9 ± 11.5, *p* < 0.01). Furthermore, unlike in IgG controls, anti-HMGB1 treatment increased animal survival (Figure 1B, 67.3 vs. 48.5%, *p* < 0.05) at day 6. The surviving anti-HMGB1-treated mice continued to appear gained weight from days 4–10 (Figure 1C, −11.9 to −3.7%, *p* < 0.05) compared to IgG controls (−22.7 to −13.6%). Indeed, instillation of anti-HMGB1 showed less interstitial congestion, inflammatory cell infiltration and proteinous exudate into the alveoli, compared to mice that received control IgG (Figures 1D,E, 1.7 ± 0.55 vs. 4.3 ± 0.68, *p* < 0.01). The lung permeability index (LPI) was significantly decreased in anti-HMGB1 group compared to IgG controls after LPS instillation (Figure 1F, 0.31 ± 0.04 vs. 0.42 ± 0.08, *p* < 0.05). As TGF-β might play an important role in the resolution of lung injury (28), we also measured its levels in BALF. We found instillation of anti-HMGB1 significantly increased TGF-β levels, as compared with controls (Figure 1G, 80.35 ± 21.74 vs. 33.3 ± 10.97, *p* < 0.01). Indeed, IgG-treated controls showed elevated levels of IL-17A, IL-23 (p19), and TNF-α in BALF (Figure 1H), whereas neutralization of HMGB1 significantly reduced these proinflammatory mediators. These results indicate that HMGB1 is crucial for triggering lung inflammation, whereas inhibition of HMGB1 promotes TGF-β yet inhibits proinflammatory cytokine programs during ALI.

### Blocking HMGB1 Inhibits PTEN but Promotes β-Catenin Activation and Tregs in Acute Lung Injury

**Figure 2:**
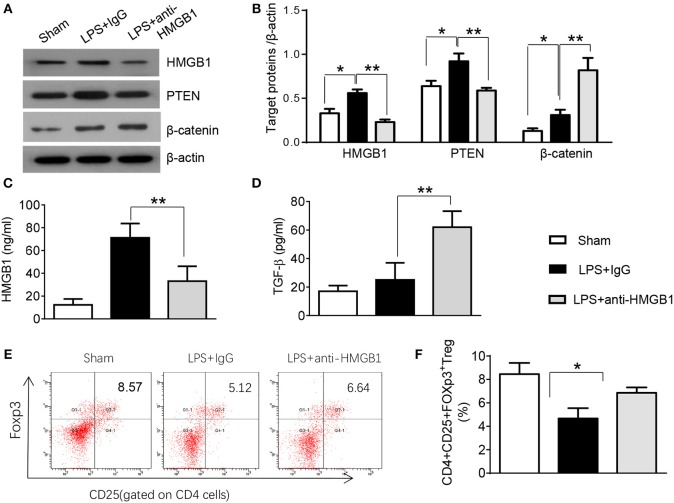
Blocking HMGB1 inhibits PTEN but promotes β-catenin activation and Tregs in acute lung injury. **(A)** The protein was isolated from alveolar macrophages in BAL fluid from sham, LPS-instilled, and anti-HMGB1 antibody-instilled mice. The expression of HMGB1, PTEN, and β-catenin was analyzed by Western blots. Representative of three experiments. **(B)** The density ratio of HMGB1, PTEN, and β-catenin. ^*^p < 0.05. ELISA assay was performed in serum from sham, LPS-instilled, and anti-HMGB1 antibody-instilled mice **(C)** HMGB1, **(D)** TGF-β, Mean ± SD (*n* = 4–6 samples/group), ^**^p < 0.01. **(E)** Representative three-dimension scatter diagrams of CD4^+^CD25^+^Foxp3^+^ Tregs in the peripheral blood from sham, LPS-instilled, and anti-HMGB1 antibody-instilled mice were analyzed by flow cytometry. **(F)** The percentage of CD4^+^CD25^+^Foxp3^+^ Tregs in the peripheral blood from sham, LPS-instilled, and anti-HMGB1 antibody-instilled mice (*n* = 4–6 animals/group), Mean ± SD, ^*^p < 0.05.

PTEN has been shown to promote inflammatory response by regulating macrophage activation (17). To test whether HMGB1 mediates PTEN activation in macrophages during lung injury, we collected alveolar macrophages from BALF after LPS instillation. Indeed, IgG control treatment significantly increased the expression of HMGB1 and PTEN in LPS-stimulated macrophages. However, blocking HMGB1 in LPS-stimulated macrophages significantly reduced PTEN and increased β-catenin expression (Figure S2, Figures 2A,B). Furthermore, HMGB1 neutralization decreased serum HMGB1 levels, as compared with controls (Figure 2C, 33.1 ± 13.1 vs. 71.2 ± 12.5, *p* < 0.01). Unlike in controls, TGF-β levels were elevated in anti-HMGB1 group (Figure 2D, 62.5 ± 11.3 vs. 25.3 ± 12.7, *p* < 0.01), accompanied by increased production of CD4^+^CD25^+^Foxp3^+^ Tregs compared to IgG controls (Figure S3, Figures 2E,F, 6.86 ± 0.46 vs. 5.67 ± 0.88, *p* < 0.05) in the peripheral blood. These results indicate LPS-induced HMGB1 activates macrophage PTEN. Upon LPS treatment, HMGB1 blockade inhibits PTEN yet promotes β-catenin activation and induction of CD4^+^CD25^+^Foxp3^+^ Tregs, which might be essential for the regulation of inflammatory response in LPS-induced ALI.

### Myeloid Cell-Specific PTEN Is Critical for the HMGB1-Mediated Inflammatory Response in Acute Lung Injury

**Figure 3:**
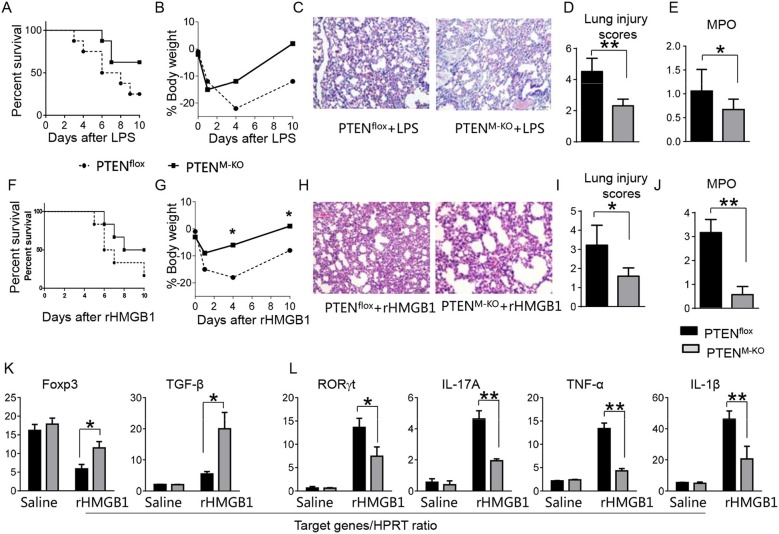
Myeloid cell-specific PTEN is critical for the HMGB1-mediated inflammatory response in acute lung injury. PTEN^flox^ and PTEN^M−KO^ Mice were given LPS or rHMGB1 at 24 h prior to lung tissue, blood, and cell harvest. **(A)** Animal survival after LPS treatment (*n* = 8 animals per group). **(B)** Body weight after LPS treatment (*n* = 8). **(C)** Lung sections from PTEN^**flox**^ and PTEN^M−KO^ mice after LPS treatment were stained with H&E. Original magnification, ×40; **(D)** Histopathological mean lung injury scores (*n* = 8); ^**^p < 0.01. **(E)** MPO assay in PTEN^flox^ and PTEN^M−KO^ mice after LPS treatment (*n* = 8 samples/group), ^**^p < 0.01. **(F)** Animal survival after rHMGB1 treatment (*n* = 8 animals per group). **(G)** Body weight after rHMGB1 treatment (*n* = 8). **(H)** Lung sections from PTEN^flox^ and PTEN^M−KO^ mice after rHMGB1 treatment were stained with H&E. Original magnification, ×40; **(I)** Histopathological mean lung injury scores (*n* = 8 animals/group); ^*^p < 0.05. **(J)** MPO assay in PTEN^flox^ and PTEN^M−KO^ mice after rHMGB1 treatment (*n* = 8 samples/group), ^**^p < 0.01. q-PCR analysis of mRNA expression coding for **(K)** Foxp3 and TGF-β, **(L)** RORγt, IL-17A, TNF-α, and IL-1β in lung tissues from PTEN^flox^ and PTEN^M−KO^ mice after rHMGB1 treatment. Mean ± SD (*n* = 4–6 samples/group), ^**^p < 0.01. Representative mean values of cytokine gene mRNA copies normalized to HPRT control.

To determine whether myeloid cell-derived PTEN plays a role in HMGB1-mediated inflammatory response during lung injury, we used myeloid cell-specific PTEN knockout (PTEN^M−KO^) mice as described (22). Indeed, increased animal survival was observed in PTEN^M−KO^ mice, but not in PTEN^flox^ control mice (Figure 3A, 83.5 vs. 46.6%, *p* < 0.01) at day 6 after LPS treatment. PTEN^M−KO^ mice exhibited weight gain (Figure 3B, −11.9 to 1.2%, *p* < 0.05) compared to controls (−22.3 to −13.5%) from days 4–10. Unlike in PTEN^flox^ controls, LPS-induced lung inflammation was attenuated in PTEN^M−KO^ mice (Figures 3C,D, 2.02 ± 0.32 vs. 3.51 ± 0.45, *p* < 0.01). Using MPO activity assay, we found decreased lung neutrophil accumulation in PTEN^M−KO^ mice after LPS stimulation, as compared with PTEN^flox^ controls (Figure 3E, 0.67 ± 0.22 vs. 1.06 ± 0.77, *p* < 0.05). Similarly, PTEN^M−KO^ increased animal survival (Figure 3F, 82.5% vs. 42.5% at day 6, *p* < 0.01) and body weight (Figure 3G, −6.5 to 1.2% vs. −18.3 to −8.8% from days 4–10, *p* < 0.01) in contrast to rHMGB1-treated PTEN^flox^ controls. Treatment of PTEN^M−KO^ mice with rHMGB1 reduced lung damage (Figures 3H,I, 2.59 ± 0.44 vs. 4.22 ± 1.27, *p* < 0.01), lung neutrophil accumulation (Figure 3J, 0.86 ± 0.45 vs. 2.55 ± 0.58, *p* < 0.005), and increased the expression of Foxp3 and TGF-β yet depressed RORγt, IL-17A, TNF-α, and IL-1β in lung tissues (Figures 3K,L, *p* < 0.01). These findings suggest that myeloid PTEN is a critical mediator for HMGB1-induced inflammatory response during ALI.

### Myeloid Cell-Specific PTEN Deficiency Activates β-Catenin Signaling and Treg Induction in Acute Lung Injury

**Figure 4:**
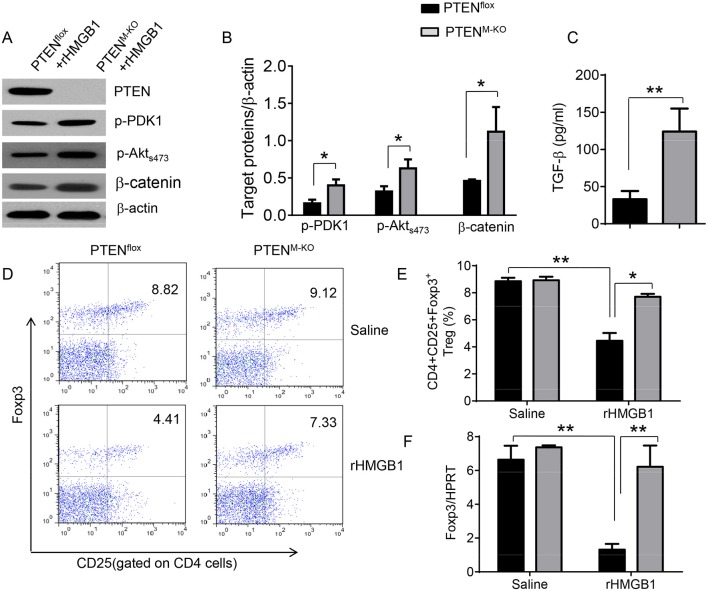
Myeloid cell-specific PTEN deficiency is critical for the induction of Tregs in acute lung injury. Mice were given rHMGB1 at 24 h prior to lung tissue, blood, and cell harvest. **(A)** The protein was isolated from peritoneal macrophages in PTEN^flox^ and PTEN^M−KO^ mice after rHMGB1 treatment. The expression of PTEN, p-PDK1, p-Akt (ser473), and β-catenin was analyzed by Western blots. Representative of three experiments. **(B)** The density ratio of p-PDK1, and p-Akt (ser473), and β-catenin. ^*^p < 0.05, ^**^p < 0.01. **(C)** ELISA-based detection of TGF-β levels in serum from PTEN^flox^ and PTEN^M−KO^ mice after rHMGB1 treatment. Mean ± SD (*n* = 4–6 samples/group), ^**^p < 0.01. **(D)** Representative diagrams of CD4^+^CD25^+^Foxp3^+^ Tregs in the peripheral blood from PTEN^flox^ and PTEN^M−KO^ mice after rHMGB1 treatment was analyzed by flow cytometry. **(E)** The percentage of CD4^+^CD25^+^Foxp3^+^ Tregs in the peripheral blood from PTEN^flox^ and PTEN^M−KO^ mice after rHMGB1 treatment (*n* = 4–6 animals/group), Mean±SD, ^*^p < 0.05. **(F)** q-PCR analysis of mRNA expression coding for Foxp3 in peripheral blood T cells from PTEN^flox^ and PTEN^M−KO^ mice after rHMGB1 treatment. Mean ± SD (*n* = 4–6 samples/group), ^**^p < 0.01.

We next test whether macrophage PTEN deficiency may affect β-catenin signaling and CD4^+^CD25^+^Foxp3^+^ Treg induction *in vivo*. We found that myeloid PTEN deficiency increased phosphorylation of PDK1 and Akt, as well as β-catenin expression in peritoneal macrophages after rHMGB1 treatment, as compared with PTEN^flox^ controls (Figures 4A,B). The serum TGF-β levels were also increased in rHMGB1-treated PTEN^M−KO^ mice compared to controls (Figure 4C, 128.7 ± 37.7 vs. 35.1 ± 15.6, *p* < 0.01). In contrast to PTEN^flox^ T cells, we observed significantly increased frequency of CD4^+^CD25^+^Foxp3^+^ Tregs (Figures 4D,E, 8.55 ± 0.77 vs. 4.61 ± 0.71, *p* < 0.05) in the peripheral blood, with substantially increased Foxp3 expression from rHMGB1-treated PTEN^M−KO^ mice (Figure 4F, *p* < 0.01). These findings implicate that macrophage PTEN deficiency can promote β-catenin signaling and CD4^+^CD25^+^Foxp3^+^ Treg induction during lung inflammatory response.

### Myeloid β-Catenin Signaling Is Essential for the Induction of CD4^+^CD25^+^Foxp3^+^ Tregs in Acute Lung Injury

**Figure 5:**
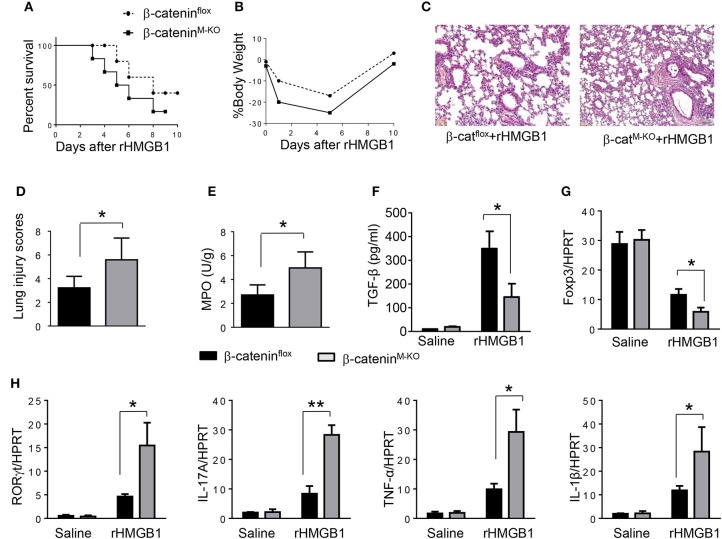
Myeloid β-catenin signaling is essential for the induction of CD4^+^CD25^+^Foxp3^+^ Treg in lung injury. The β-catenin^**flox**^ and β-catenin^M−KO^ mice were given rHMGB1 at 24 h prior to lung tissue and blood harvest. **(A)** Animal survival after rHMGB1 treatment (*n* = 8 animals per group). **(B)** Body weight after rHMGB1 treatment (*n* = 8). **(C)** Lung sections from β-catenin^flox^ and β-catenin^M−KO^ mice after rHMGB1 treatment were stained with H&E. Original magnification, ×40. **(D)** Histopathological mean lung injury scores (*n* = 8); ^**^p < 0.01. **(E)** MPO assay in β-catenin^flox^ and β-catenin^M−KO^ mice after rHMGB1 treatment (*n* = 8 samples/group). **(F)** ELISA-based detection of TGF-β in serum from β-catenin^flox^ and β-catenin^M−KO^ mice after rHMGB1 treatment. Mean±SD (*n* = 4–6 samples/group). q-PCR analysis of mRNA expression coding for **(G)** Foxp3 and **(H)** RORγt, IL-17A, TNF-α, and IL-1β in lung tissues from β-catenin^flox^ and β-catenin^M−KO^ mice after rHMGB1 treatment. Mean ± SD (*n* = 4–6 samples/group). Representative mean values of cytokine gene mRNA copies normalized to HPRT control. ^*^p < 0.05, ^**^p < 0.01.

**Figure 6:**
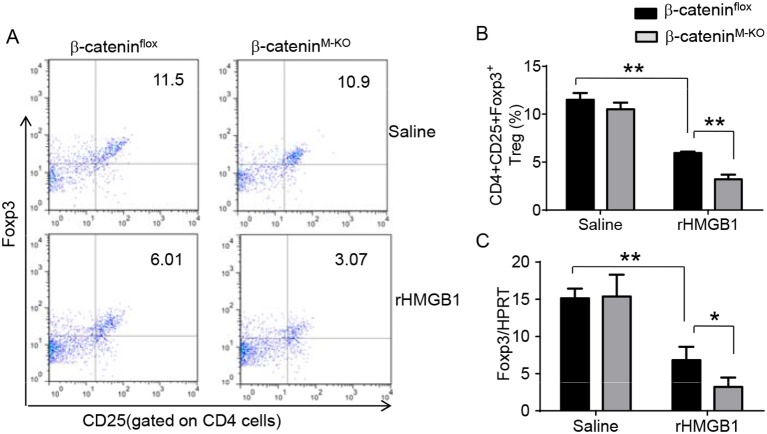
Myeloid β-catenin deficiency reduces the frequency of CD4^+^CD25^+^Foxp3^+^ Tregs and Foxp3 expression in lung injury. The β-catenin^flox^ and β-catenin^M−KO^ mice were given rHMGB1 at 24 h prior to blood harvest. **(A)** Representative diagrams of CD4^+^CD25^+^Foxp3^+^ Tregs in the peripheral blood from β-catenin^flox^ and β-catenin^M−KO^ mice after rHMGB1 treatment was analyzed by flow cytometry. **(B)** The percentage of CD4^+^CD25^+^Foxp3^+^ Tregs from β-catenin^flox^ and β-catenin^M−KO^ mice after rHMGB1 treatment (*n* = 4–6 animals/group), Mean ± SD, ^*^p < 0.05. **(C)** q-PCR analysis of mRNA expression coding for Foxp3 in peripheral blood T cells from β-catenin^flox^ and β-catenin^M−KO^ mice after rHMGB1 treatment. Mean ± SD (*n* = 4–6 samples/group), ^**^p < 0.01.

To determine the role of β-catenin activation in producing CD4^+^CD25^+^Foxp3^+^ Tregs, we used myeloid cell-specific β-catenin knockout (β-catenin^M−KO^) mice. Indeed, animal survival rate was decreased in β-catenin^M−KO^ mice, but not in β-catenin^flox^ control mice (Figure 5A, 28.5 vs. 58.2%, *p* < 0.01) at day 6 after rHMGB1 treatment. The body weight was decreased in β-catenin^M−KO^ mice (Figure 5B, −25.5 to 3.2%, *p* < 0.05) compared to controls (−12.4 to 3.5%) from days 4–10. Unlike in β-catenin^flox^ controls, rHMGB1 treatment exacerbated lung injury in β-catenin^M−KO^ mice (Figures 5C,D, 3.22 ± 0.98 vs. 5.59 ± 1.84, *p* < 0.05). MPO activity assay displayed an increased lung neutrophil accumulation in β-catenin^M−KO^ mice after rHMGB1 treatment, as compared with β-catenin^flox^ controls (Figure 5E, 4.97 ± 1.34 vs. 2.7 ± 0.58, *p* < 0.05). rHMGB1 treatment in β-catenin^M−KO^ mice decreased TGF-β release (Figure 5F, *p* < 0.001) and Foxp3 expression (Figure 5G, *p* < 0.05) and yet augmented RORγt, IL-17A, TNF-α, and IL-1β (Figure 5H, *p* < 0.05) in lung tissues. Moreover, a reduced frequency of CD4^+^CD25^+^Foxp3^+^ Tregs (Figures 6A,B, *p* < 0.01), accompanied by decreased Foxp3 expression in the peripheral blood (Figure 6C, *p* < 0.05) was observed in β-catenin^M−KO^ mice after rHMGB1 treatment. These findings implicate that macrophage β-catenin deficiency reduces TGF-β release, Foxp3 expression, and CD4^+^CD25^+^Foxp3^+^ Treg induction while increasing RORγt/IL-17A, implying the essential role of β-catenin in the mechanism of CD4^+^CD25^+^Foxp3^+^ Treg induction during lung inflammatory response.

### Disruption of the HMGB1-PTEN Axis Promotes β-Catenin Signaling and Induces Tregs *in vitro*

**Figure 7:**
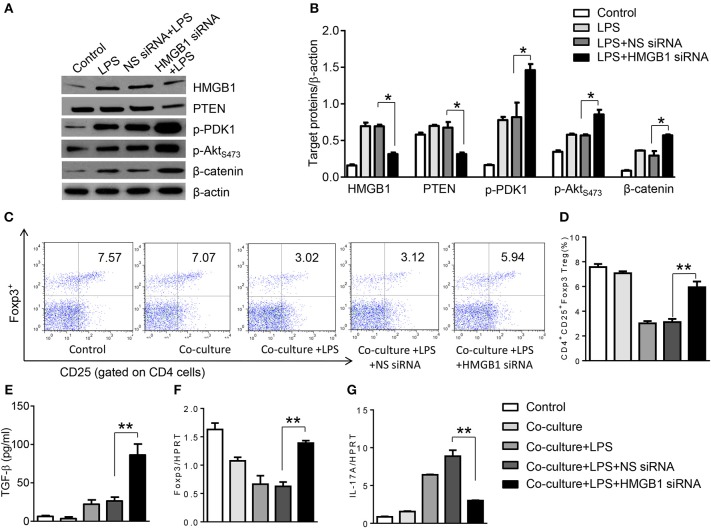
HMGB1 blockade induces PDK1/Akt/β-catenin activation and CD4^+^CD25^+^Foxp3^+^ Tregs *in vitro*. The peritoneal macrophages from WT mice was transfected with HMGB1 siRNA or NS siRNA, and then co-cultured with spleen T cells after LPS stimulation for 6 h. **(A)** The protein was isolated from HMGB1 siRNA- or NS siRNA-transfected macrophages in the co-cultures. The expression of PTEN, p-PDK1, p-Akt (ser473), and β-catenin was analyzed by Western blots. Representative of three experiments. **(B)** The density ratio of HMGB1, PTEN, p-PDK1, p-Akt (ser473), and β-catenin. ^*^p < 0.05, ^**^p < 0.01. **(C)** Representative diagrams of CD4^+^CD25^+^Foxp3^+^Tregs in spleen T cells from co-cultures was analyzed by flow cytometry. **(D)** The percentage of CD4^+^CD25^+^Foxp3^+^ Tregs in spleen T cells from co-cultures (*n* = 4–6 samples/group), Mean ± SD, ^**^p < 0.01. **(E)** ELISA analysis of TGF-β levels in the supernatants from co-cultures. Mean ± SD (*n* = 4–6 samples/group). q-PCR analysis of mRNA expression coding for **(F)** Foxp3 and **(G)** IL-17A in spleen T cells from co-cultures. Mean ± SD (*n* = 4–6 samples/group), ^*^p < 0.05, ^**^p < 0.01.

**Figure 8:**
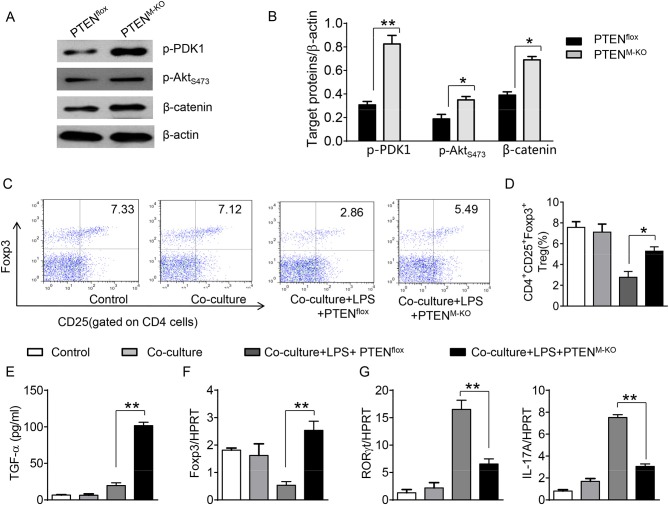
Myeloid PTEN deficiency promotes β-catenin signaling and induces CD4^+^CD25^+^Foxp3^+^ Tregs *in vitro*. The peritoneal macrophages were isolated from PTEN^flox^ and PTEN^M−KO^ mice, and then co-cultured with spleen T cells after LPS stimulation for 6 h. **(A)** The protein was isolated from macrophages in the co-cultures. The expression of p-PDK1, p-Akt (ser473), and β-catenin was analyzed by Western blots. Representative of three experiments. **(B)** The density ratio of p-PDK1, and p-Akt (ser473), β-catenin. ^*^p < 0.05. **(C)** Representative diagrams of CD4^+^CD25^+^Foxp3^+^Tregs in spleen T cells from co-cultures was analyzed by flow cytometry. **(D)** The percentage of CD4^+^CD25^+^Foxp3^+^ Tregs in spleen T cells from co-cultures (*n* = 4–6 samples/group), Mean ± SD, ^**^p < 0.01. **(E)** ELISA analysis of TGF-β levels in the supernatants from co-cultures. Mean±SD (*n* = 4–6 samples/group), ^*^p < 0.05. q-PCR analysis of mRNA expression coding for **(F)** Foxp3, **(G)** RORγt and IL-17A in spleen T cells from co-cultures. Mean ± SD (*n* = 4–6 samples/group), ^*^p < 0.05, ^**^p < 0.01.

**Figure 9:**
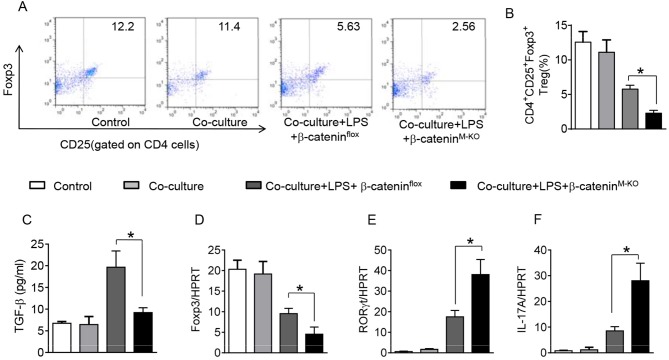
Myeloid β-catenin signaling is essential for the induction of CD4^+^CD25^+^Foxp3^+^ Tregs *in vitro*. The peritoneal macrophages were isolated from β-catenin^flox^ and β-catenin^M−KO^ mice, and then co-cultured with spleen T cells after LPS stimulation for 6 h. **(A)** Representative diagrams of CD4^+^CD25^+^Foxp3^+^Tregs in spleen T cells from co-cultures was analyzed by flow cytometry. **(B)** The percentage of CD4^+^CD25^+^Foxp3^+^ Tregs in spleen T cells from co-cultures (*n* = 4–6 samples/group), Mean ± SD. **(C)** ELISA analysis of TGF-β levels in the supernatants from co-cultures. Mean ± SD (*n* = 4–6 samples/group), ^*^p < 0.05. q-PCR analysis of mRNA expression coding for **(D)** Foxp3, **(E)** RORγt, and IL-17A **(F)** in spleen T cells from co-cultures. Mean ± SD (*n* = 4–6 samples/group), ^*^p < 0.05.

To further elucidate the potential mechanisms of the macrophage HMGB1/PTEN/β-catenin signaling in mediating Tregs during lung injury, we used the macrophage/spleen T cell co-culture system. We blocked HMGB1 with siRNA transfection in LPS-stimulated macrophages, and then co-cultured with spleen T cells. Indeed, HMGB1 knockdown decreased macrophage PTEN yet augmented p-PDK1, p-Akt, and β-catenin as compared with the NS siRNA-treated controls (Figures 7A,B). Staining spleen T cells from co-cultures by flow cytometry revealed significantly increased percentage of CD4^+^CD25^+^Foxp3^+^ Tregs in HMGB1 siRNA-transfected cultures, compared to siRNA-treated controls (Figures 7C,D, 5.94 ± 0.55 vs. 3.12 ± 0.38, *p* < 0.01). Moreover, the HMGB1 knockdown significantly increased TGF-β levels in co-culture supernatants, compared to NS siRNA-treated controls (Figure 7E, 88.5 ± 26.3 vs. 26.8 ± 11.8, *p* < 0.01). Unlike control cultures, the Foxp3 expression was significantly increased, whereas IL-17A expression was suppressed in spleen T cells from HMGB1 siRNA-transfected co-cultures (Figures 7F,G). Furthermore, in contrast to PTEN^flox^ controls, macrophage PTEN deficiency increased the expression of p-PDK1, p-Akt, and β-catenin (Figures 8A,B), accompanied by markedly increased percentage of CD4^+^CD25^+^Foxp3^+^ Tregs (Figures 8C,D, 5.3 ± 0.43 vs. 2.7 ± 0.33, *p* < 0.05), TGF-β levels (Figure 8E, 94.6 ± 12.6 vs. 18.3 ± 4.5, *p* < 0.01), and Foxp3 (Figure 8F) yet reduced RORγt and IL-17A expression (Figure 8G) in PTEN-deficient co-cultures. To confirm the importance of β-catenin signaling in the production of CD4^+^CD25^+^Foxp3 Tregs, we further analyzed the frequency of CD4^+^CD25^+^Foxp3^+^ Tregs in spleen T cells after co-culturing with β-catenin-deficient macrophages. Indeed, a decreased percentage of CD4^+^CD25^+^Foxp3^+^ Tregs (Figures 9A,B, *p* < 0.05), with substantially reduced TGF-β levels (Figure 9C, *p* < 0.05) and Foxp3 expression (Figure 9D, *p* < 0.05), yet increased RORγt and IL-17A expression (Figures 9E,F, *p* < 0.05) was observed in β-catenin^M−KO^ co-cultures, as compared with β-catenin^flox^ controls. Taken together, these findings indicate a potential mechanism by which disruption of HMGB1/PTEN axis activates β-catenin signaling and promotes TGF-β, which contributes to the induction of CD4^+^CD25^+^Foxp3^+^ Tregs during lung injury in sepsis.

## Discussion

In this study, we have demonstrated, for the first time, that the HMGB1/PTEN/β-catenin signaling represents a novel regulatory pathway to induce CD4^+^CD25^+^Foxp3^+^ Tregs in sepsis-induced lung injury.

Using the animal model of ALI, we found instillation of LPS triggered systemic inflammatory response and induced ALI, which was accompanied by induction of HMGB1. Though the exacerbated lung damage was shown in LPS instilled lungs, neutralization of HMGB1 with anti-HMGB1 antibody provided significant protection against ALI as evidenced by increasing animal survival and decreasing pulmonary edema. These findings are consistent with previous reports that intratracheal instillation of live bacterial or HMGB1 mediates an acute inflammatory response characterized by the development of pulmonary edema and increased intrapulmonary production of proinflammatory cytokines (8, 17, 29).

Numerous studies have revealed the ability of CD4^+^CD25^+^Foxp3^+^ Tregs to control immune responses in lung injury (13, 30–32). In a mouse model of LPS-induced ALI, we found that increased HMGB1 levels mitigated the accumulation of CD4^+^CD25^+^Foxp3^+^ Tregs leading to exacerbated lung damage. Interestingly, increasing HMGB1 release and protein expression enhanced PTEN activation on alveolar macrophages after LPS instillation. However, neutralization of HMGB1 suppressed PTEN, which was accompanied by increased CD4^+^CD25^+^Foxp3^+^ Tregs and reduced IL-17A in LPS-treated mice. Consistent with previous reports that deletion of PTEN enhanced the expansion of CD4^+^CD25^+^Tregs (33), our results indicate that PTEN might serve as a negative regulator of Treg peripheral homeostasis during lung inflammation.

Further evidence of PTEN-mediated modulation of Tregs in ALI was obtained from myeloid cell-specific PTEN knockout (PTEN^M−KO^) mice. We found that, in contrast to the PTEN^**flox**^ mice, PTEN^M−KO^ mice treated with LPS or rHMGB1 had reduced lung injury, neutrophil accumulation, proinflmmatory mediators, and increased animal survival. Moreover, myeloid PTEN deficiency increased β-catenin expression and phosphorylation of PDK1 and Akt on macrophages, accompanied by increased peripheral Tregs and Foxp3 expression yet decreased RORγt and IL-17A. Since increasing release of HMGB1 induced macrophage PTEN activation, while deleting myeloid PTEN promoted Tregs, we believe that PTEN is a mediator in the modulation of innate and adaptive immunity during lung inflammation. Indeed, alveolar macrophages are essential for the initiation of innate immune response by binding the toll-like receptors (TLRs) (34). In response to TLRs, PTEN activation on macrophages triggers inflammatory response via regulating PI3K signaling (35, 36). Notably, our current data demonstrated that myeloid PTEN deficiency promoted β-catenin activation, consistent with our previous report that PTEN-mediated β-catenin signaling regulated Foxo1-TLR4 activation in lung inflammation (37), suggesting the endogenous innate immune signaling most likely contributes to the Treg induction. Indeed, expression of stabilized β-catenin controls Treg development and survival (38). Activation of β-catenin regulates inflammatory response and promotes anti-inflammatory mediator (39). Thus, our findings implicate that disruption of macrophage HMGB1 or PTEN, and activation of β-catenin may be a key pathway in the regulation of Treg development during lung injury.

The mechanisms underlying the macrophage HMGB1/PTEN/β-catenin signaling-mediated Treg induction appear to be complex during ALI. Our data showed that HMGB1 blockade or PTEN loss increased TGF-β release. However, reduced TGF-β release was observed from β-catenin deficient-macrophages in response to rHMGB1 stimulation. This is consistent with previous report that β-catenin was required for the TGF-β production to regulate immunity during inflammatory response (39). Indeed, TGF-β is a potent regulator of the immune and inflammatory system. *In vitro* stimulation of naïve CD4^+^ T cells in the presence of TGF-β increased the expression of CD4^+^CD25^+^Foxp3^+^ associated with *in vivo* suppressive activity during lung inflammatory response (40). Disruption of TGF-β impaired the development of Foxp3^+^ Tregs and may lead to the multifocal inflammatory cell infiltration and multiorgan failure in mice (28, 41). Moreover, TGF-β inhibited RORγt activity and Th17 cell differentiation in human CD4^+^ T cells (42). TGF-β-induced Foxp3 inhibited Th17 cell differentiation by regulating RORγt function (43). TGF-β promoted the development of Treg and expansion Foxp3^+^-expressing CD4^+^CD25^+^ Tregs *in vivo* (44, 45). Lung-resident tissue macrophages can generate Foxp3^+^ Tregs through increasing TGF-β expression (46). Consistent with this notion, we found increased TGF-β expression and secretion by alveolar macrophages were accompanied by increased CD4^+^CD25^+^Foxp3^+^ Tregs and reduced RORγt/IL-17A after anti-HMGB1 treatment or myeloid PTEN deletion in our animal models. This implies that TGF-β may be essential for the induction of CD4^+^CD25^+^Foxp3^+^ Tregs during HMGB1-induced inflammatory response. On the other hand, we found HMGB1 knockdown markedly inhibited macrophage PTEN expression in the co-culture system. This is consistent with deletion of myeloid PTEN, which increased the expression of PDK1, Akt, and β-catenin. Although PTEN deficiency increased the frequency of CD4^+^CD25^+^Foxp3^+^ Tregs, ablation of myeloid β-catenin resulted in reduced CD4^+^CD25^+^Foxp3^+^ Tregs and increased RORγt/IL-17A. Indeed, our previous study has shown that disruption of PTEN increased β-catenin, which in turn promoted PI3K/Akt signaling to native feedback to regulate TLR4-driven inflammatory response (47). Increased β-catenin activity enhanced TGF-β production on macrophages, whereas β-catenin deficiency lost the ability to produce TGF-β, myeloid cell motility and adhesion leading to impairing tissue repair (48). Hence, the HMGB1/PTEN/β-catenin signaling regulates Treg induction through multiple signaling pathways. Recent works indicated that PDK1, a downstream of PI3K signaling, plays an important role in the regulation of Treg function (21). PDK1 deficiency suppressed Treg accumulation while increasing IL-17-expressing population leading to enhancing inflammatory response (21). Activation of Akt by PDK1 phosphorylation promoted Tregs and enhanced their suppressive capacity to the Th17 cell differentiation (20). Furthermore, increased Akt phosphorylation enhanced β-catenin transcriptional activity (49). Activation of β-catenin is essential for the stimulation of Treg induction while inhibition of inflammatory T cells (39). These data are consistent with our results that activation of PDK1/Akt/β-catenin enhanced Treg induction and suppressed IL-17A transcription regulated by RORγt *in vitro* and *in vivo*. Although our current study was based on the primary ALI and it might have some modified signaling pathways with secondary ALI (systemic inflammation), our findings suggest that HMGB1/PTEN/β-catenin signaling is critical to contribute to the induction of CD4^+^CD25^+^Foxp3^+^ Tregs in sepsis-induced lung injury.

In the present study, we observed that HMGB1 can be induced in endotoxin-stimulated macrophages during sepsis. HMGB1 induction activates PTEN and inhibits PI3K/PDK1/Akt leading to suppressed β-catenin activity, which then decreases TGF-β release from macrophages, results in diminished Foxp3^+^ Treg induction. Blockade of HMGB1 or macrophage PTEN deletion activates PI3K/PDK1/Akt and β-catenin signaling, which in turn enhances macrophage TGF-β leading to increased Foxp3 Treg induction while inhibiting Th17 cell differentiation during sepsis-induced lung injury.

In conclusion, the macrophage HMGB1/PTEN/β-catenin signaling displays a distinct capacity to regulate the development of CD4^+^CD25^+^Foxp3^+^ Tregs during lung inflammation. Induction of Tregs ultimately alleviated inflammatory response and facilitated resolution of lung injury. By identifying the regulatory pathway of HMGB1/PTEN/β-catenin signaling on Treg induction, our studies provide the rationale for novel therapeutic strategies for treating sepsis-induced lung injury.

## Data Availability

The raw data supporting the conclusions of this manuscript is available, without undue reservation, to any qualified researcher.

## Ethics Statement

The animal study was performed in strict accordance with the recommendations in the Guide for the Care and Use of Laboratory Animals published by the National Institutes of Health. The study protocol were approved by the Institutional Animal Care and Use Committee of Anhui Medical University (No: LLSC2013007).

## Author Contributions

MZ contributed to the experimental design, performed research, analyzed data, and wrote the first draft of manuscript. MD, RT, HL, ZG, and ZJ collected and analyzed the human samples. HF wrote and revised the manuscript. CL performed *in vitro* experiments. X-LC and BK contributed to the study concept, research design, data analysis, and finalized the manuscript.

### Conflict of Interest Statement

The authors declare that the research was conducted in the absence of any commercial or financial relationships that could be construed as a potential conflict of interest.
