# Supplementary material for: The Modulation of Regulatory T Cells via HMGB1/PTEN/β-Catenin Axis in LPS Induced Acute Lung Injury
Source: Front Immunol. 2019 Jul 25;10:1612. doi: 10.3389/fimmu.2019.01612 (PMC6669370; doi:10.3389/fimmu.2019.01612)
Supplement: Supplementary file 1 [file Data_Sheet_1.docx]

| **Supplementary Table 1**: Primer sequences for the amplification | | |
| --- | --- | --- |
| Target genes | Forward primers | Reverse primers |
| HPRT | 5’-TCAACGGGGGACATAAAAGT-3’ | 5’-TGCATTGTTTTACCAGTGTCAA-3’ |
| TNF-α | 5’-GCCTCTTCTCATTCCTGCTTGT-3’ | 5’-GATGATCTGAGTGTGAGGGTCTG-3’ |
| TGF-β | 5’-GCAACATGTGGAACTCTACCAG-3’ | 5’-CAGCCACTCAGGCGTATCA-3’ |
| IL-17A | 5’- ACTACCTCAACCCTTCCA-3’ | 5’- GCTTCCCAGATCACAGAG-3’ |
| IL-23  RORγt  Foxp3 | 5’- ACTAAGAGAAGAAGAGGAT-3’  5’-AGAGACACCACCGGACATCT-3’  5’- AGCTCCTCTGCCGTTATCC-3’ | 5’- GAAGATGTCAGAGTCAAG-3’  5’- CAAGGGATCACTTCAATTTGTG-3’  5’- GCAAGACTCCTGGGGATG-3’ |
