# Supplementary material for: The Modulation of Regulatory T Cells via HMGB1/PTEN/β-Catenin Axis in LPS Induced Acute Lung Injury
Source: Front Immunol. 2019 Jul 25;10:1612. doi: 10.3389/fimmu.2019.01612 (PMC6669370; doi:10.3389/fimmu.2019.01612)

Figure S1. Expression of β-catenin was detected in myloid cells and spleens of β-catenin^FL/FL^ andβ-catenin^M-KO^ mice by using western blot.


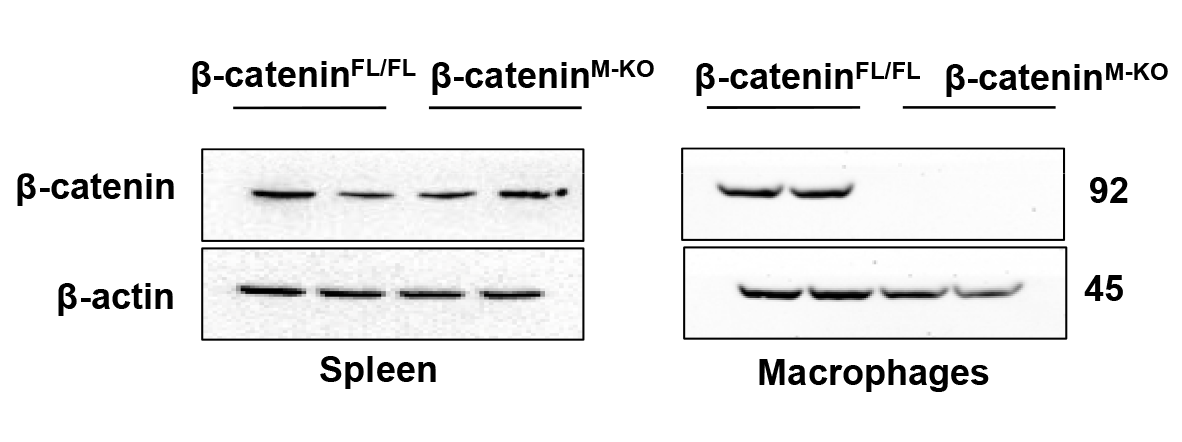


Figure S2. Expression of PTEN, β-catenin, p-Akt after rHMGB1 treatment was detercted by using western blot (A), and The density ratio of PTEN, β-catenin, and p-Akt to β-actin (B).


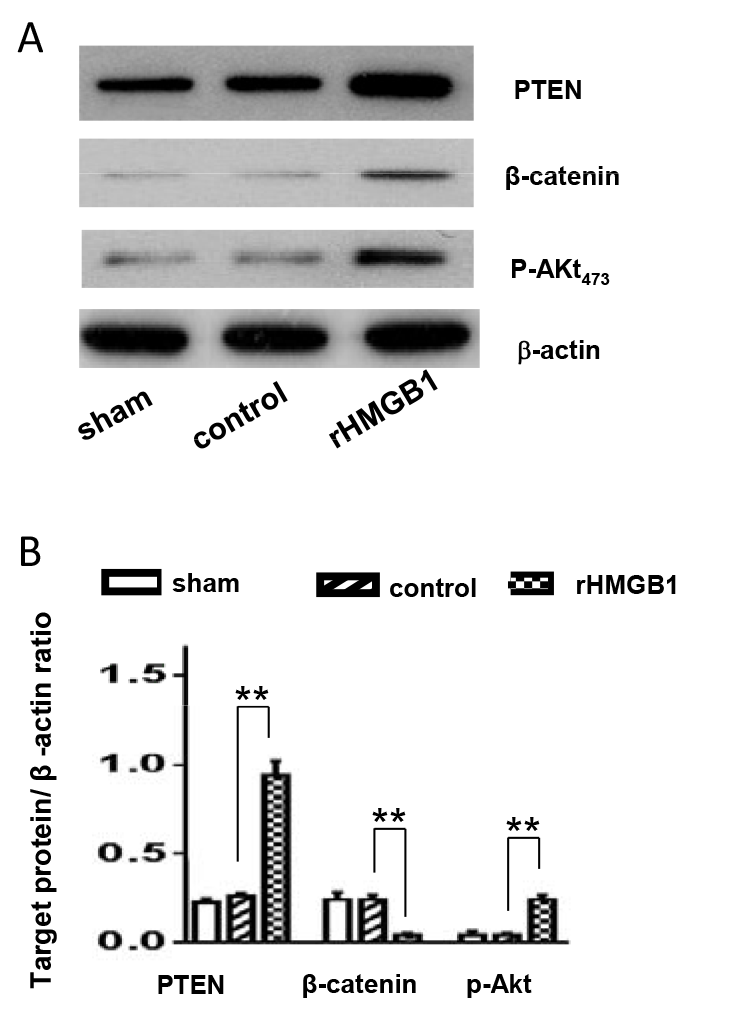


Figure S3. Isotype control of the cytokines staining.


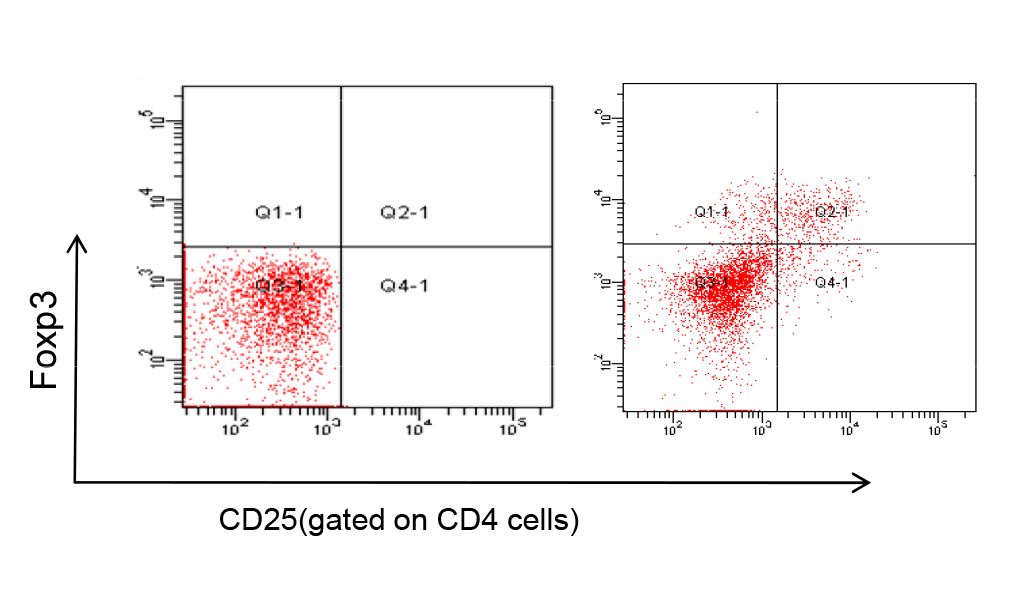

Supplement: Supplementary file 2 [file Data_Sheet_2.doc]
